# The biological responses of vitamin K2: A comprehensive review

**DOI:** 10.1002/fsn3.3213

**Published:** 2023-01-06

**Authors:** Quanxiang Yan, Tao Zhang, Christine O'Connor, James W. Barlow, John Walsh, Gaia Scalabrino, Feng Xu, Helen Sheridan

**Affiliations:** ^1^ Institute of Science and Technology Shenyang Open University Shenyang China; ^2^ School of Food Science & Environmental Health Technological University Dublin Dublin 7 Ireland; ^3^ NatPro Centre, School of Pharmacy and Pharmaceutical Sciences Trinity College Dublin Dublin 2 Ireland; ^4^ Department of Chemistry RCSI University of Medicine and Health Sciences Dublin 2 Ireland; ^5^ School of Pharmacy and Pharmaceutical Sciences Trinity College Dublin Dublin 2 Ireland; ^6^ The Centre of Vitamin K2 Research Shenyang Pharmaceutical University Shenyang China

**Keywords:** biological response, menaquinones, supplementation, vitamin K1 (VitK1), vitamin K2 (VitK2)

## Abstract

Vitamin K1 (VitK1) and Vitamin K2 (VitK2), two important naturally occurring micronutrients in the VitK family, found, respectively, in green leafy plants and algae (VitK1) and animal and fermented foods (VitK2). The present review explores the multiple biological functions of VitK2 from recently published *in vitro* and *in vivo* studies, including promotion of osteogenesis, prevention of calcification, relief of menopausal symptoms, enhancement of mitochondrial energy release, hepato‐ and neuro‐protective effects, and possible use in treatment of coronavirus disease. The mechanisms of action associated with these biological effects are also explored. Overall, the findings presented here suggest that VitK, especially VitK2, is an important nutrient family for the normal functioning of human health. It acts on almost all major body systems and directly or indirectly participates in and regulates hundreds of physiological or pathological processes. However, as biological and clinical data are still inconsistent and conflicting, more in‐depth investigations are warranted to elucidate its potential as a therapeutic strategy to prevent and treat a range of disease conditions.

## Graphical abstract

This paper provides insight into the potential beneficial roles of vitamin K2 in human health by reviewing, comparing, and contrasting available *in vitro*, *in vivo*, and clinical data. This review also provides a guidance to agencies to review the current recommendation of vitamin K2 daily intake as a supplement.

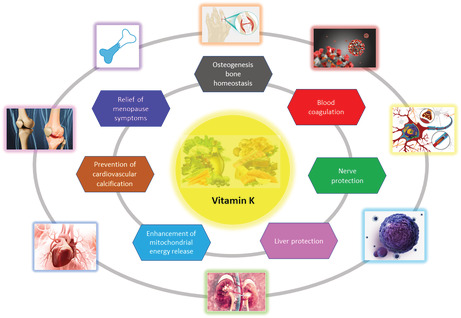

## 1 INTRODUCTION

**FIGURE 1:**
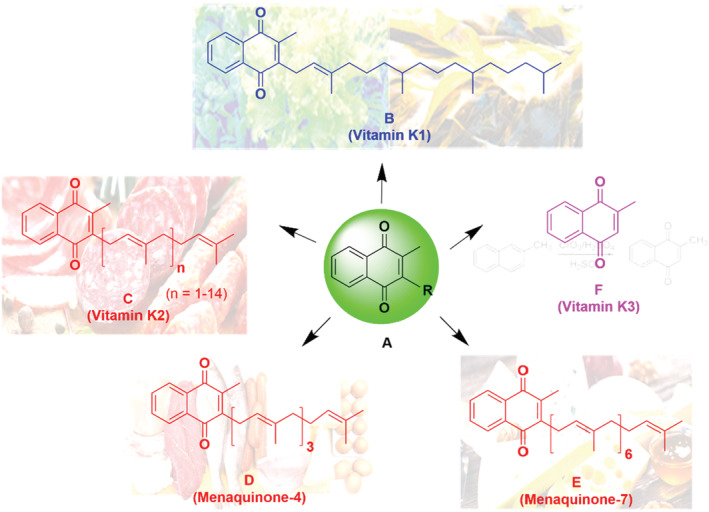
Different types of vitamin K. (a) The common methylated naphthoquinone scaffold; (b) Phylloquinone (vitamin K1, sources: plants and algae); (c) Menaquinone‐n, varying in length up to menaquinone‐15 (MK‐15) (vitamin K2, sources: animal foods and fermented products); (d) MK‐4; E. MK‐7; F. Menadione (vitamin K3, a synthetic compound).

Vitamin K (VitK) is a family of lipid‐soluble molecules first discovered in 1929 (Dam, 1935). Since then, VitK has been widely studied, both chemically and biologically. VitK presents in three subtypes, VitK1 (phylloquinone), VitK2 (menaquinones), and VitK3 (menadione), each containing the common core structure of a methylated naphthoquinone (known as 2‐methylnaphthalene‐1,4‐dione) scaffold (Figure 1). Both VitK1 and VitK2 are naturally occurring, found either in green leafy plants and algae (via photosynthesis) or in animal and fermented foods (predominantly via certain bacterial synthesis), respectively (Conly et al., 1994).

Vitamin K2 comprises a collection of different compounds known as menaquinone‐n (MK‐n), with variation in length of the unsaturated isoprene side chain in the molecule (*n*: number of isoprene units; Figure 1), ranging from MK‐2 to MK‐15 according to Kurosu and Begari (2010). MK‐4, MK‐7, and MK‐9 are the most studied menaquinones, especially MK‐4. Anaerobic bacteria in the colon can convert MK‐10 to MK‐13. MK‐4, alone among the different menaquinones, may be produced in the body via a conversion process from VitK1 or VitK3, without the involvement of bacterial action (Suttie, 2012), yet VitK2 is considered to be of animal origin based on its tissue‐specific conversion from phylloquinone (VitK1) (Nakagawa et al., 2010). There is also evidence indicating that MK‐4 can be converted from some MKs with longer side chains (e.g., MK‐7; Schurgers et al., 2006). MK‐4 has the highest bioactivity in the VitK2 category, although MK‐7 possesses higher bioavailability and a longer half‐life due to its more hydrophobic nature (Akbari & Rasouli‐Ghahroudi, 2018). The richest source of MK‐7 has been found to be ‘natto’, a Japanese food made from soybeans, which are fermented with *Bacillus subtilis* subspecies *natto* BEST195 (Kaneki et al., 2001). The majority of bodily VitK1 is stored in the liver, while MK‐4 is the primary VitK form in humans. It is found in diverse organs, including the brain, pancreas, and genital organs (Akbari & Rasouli‐Ghahroudi, 2018). VitK3, a synthetic form of VitK, is widely used in animal husbandry, but needs to be converted to MK‐4 to be active (Nakagawa et al., 2010) and is rarely used to treat VitK deficiency due to potential toxicity issues (Ren et al., 2020). In a recent review, the authors reviewed the dietary sources and production methods of VitK. They also compared and contrasted the function, absorption, storage, and bioavailability of VitK1 and VitK2 (Simes et al., 2020).

This paper primarily and critically reviews the biological responses of VitK2, with the aim of clarifying the potential mechanistic pathways associated with its observed bioactivities, and of exploring its usefulness as a potential therapeutic strategy for various diseases. Clarification of the role of VitK2 in disease prevention and health maintenance is increasingly important, especially in light of the global pandemic, caused by the coronavirus disease 2019 (COVID‐19) virus, where there is a need for suitable and effective treatments and approaches to boosting general population health.

Methods used to formulate this article included review of published reports, gathered from the following databases: PubMed, Scopus, ISI Web of Science, and CNKI. The electronic search was initially performed from inception to 30 November 2021 and updated on 30 June 2022, without language restrictions. We considered original research articles, including experimental, observational, or clinical trials. The publications were retrieved using the search terms and text words: “vitamin K2” or “menaquinone” in combination with “health” or “diseases”. The database search was supplemented by consulting the bibliography of the articles, reviews, and published meta‐analyses. The literature research was not limited to a time period, but a particular focus was given to the studies from the past 20 years. Relevant articles were chosen after reviewing through all titles and abstracts, and full texts were obtained if the information contained in the title or abstract was insufficient to exclude the study. As the nature of the research in this area has been largely animal studies and case reports, we did not make a formal assessment of the quality of the research or undertake a formal systematic review with meta‐analysis of the quality of evidence for those studies. When the forms of VitK (between VitK1 and VitK2) used were not specified, those studies were excluded unless the outcomes of the studies were considered to be important to report, such as the studies on COVID‐19.

## 2 BIOLOGICAL RESPONSE TO VITAMIN K2: MAIN FINDINGS AND DISCUSSION

**FIGURE 2:**
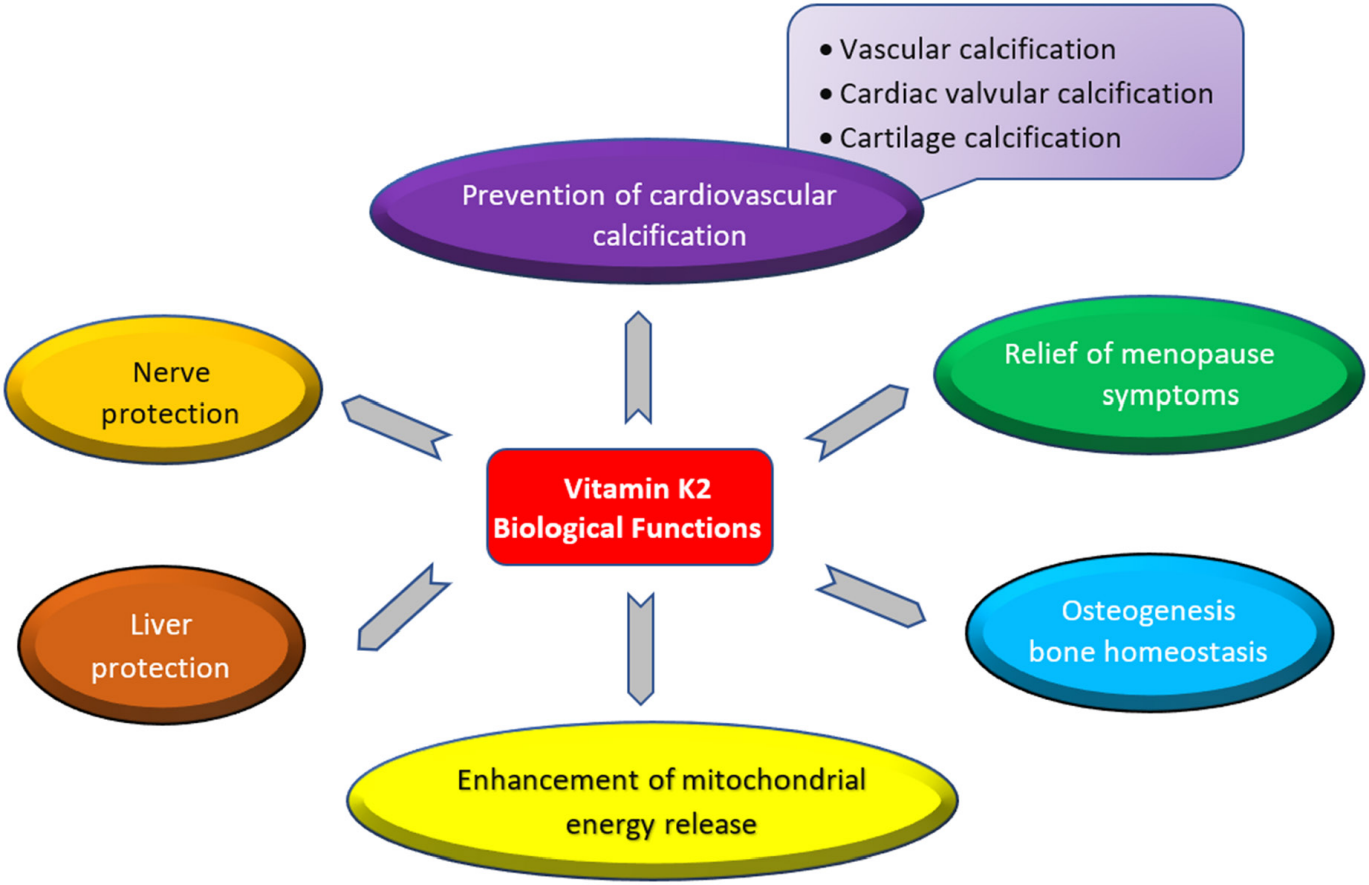
Biological responses of vitamin K2.

To explore the biological activities of VitK2 at multiple levels, extensive *in vitro/in vivo* studies and hundreds of clinical observations have been performed. Such studies have included, amongst others, morphological, biochemical, immunohistochemical, biometric, biomechanical, and molecular biological approaches. There is accumulating evidence that VitK2 acts on almost every system in the body, and has diverse roles through direct or indirect participation in, and regulation of hundreds of physiological and pathological processes (Beulens et al., 2013). There is a body of evidence showing that such wide involvement of VitK2 is directly linked to calcium homeostasis, which participates in various physiological processes, and that the main role of VitK2 is to maintain a steady level of calcium in multiple biological pathways (Okano, 2016). Various aspects of these activities of VitK2 are discussed in this section (Figure 2), and Table 1 summarizes the studies discussed in this review linking VitK2 and health. Since its role in maintaining normal blood coagulation has been thoroughly documented as VitK's most well‐known function, this bioactivity is not covered in this review.

**TABLE 1:** Experimental evidence linking vitamin K2 (VitK2) and health.

Study type	Type of pathology	Key findings	References
*In vitro*	AD	VitK2 possesses antiapoptotic and antioxidant effects and maybe a valuable protective candidate against the progression of AD via inactivating p38 MAP kinase pathway	Hadipour et al. (2020)
	AD	VitK2 protects neural cells against Aβ‐toxicity probably via regulating PI3K associated‐signaling pathway and inhibiting caspase‐3‐mediated apoptosis	Huang et al. (2021)
	AD & mitochondrial dysfunction	VitK2 modulates mitochondrial dysfunction induced by 6‐hydroxydopamine in SH‐SY5Y cells via mitochondrial quality‐control loop through the Pink1/Parkin signaling pathway	Tang et al. (2022)
	Arterial calcification	VitK2 and pamidronate synergistically inhibit arterial calcification via the increased expression of tropoelastin	Saito et al. (2007)
	Astrocyte dysfunction	VitK2 decreases hypoxia‐induced damage of astrocytes, provides high astrocyte activity, and reduces the production of ROS and superoxide oxide, with possible involvement of Gas6 and protein S	Yang et al. (2017)
	Bone resorption & homeostasis	MK inhibits bone resorption partly by suppressing prostaglandin E2 synthesis	Hara et al. (1995)
	Bone resorption & homeostasis	VitK2 acts as a transcriptional regulator of extracellular matrix‐related genes during bone formation	Ichikawa et al. (2006)
	Coenzyme Q_10_ deficiency	VitK2 cannot substitute Coenzyme Q_10_ as electron carrier in the mitochondrial respiratory chain of mammalian cells; hence, it could not restore either electron flow or ATP biosynthesis in Coenzyme Q_10_‐deficient cells	Cerqua et al. (2019)
	HCC	VitK2 inhibits MMP expression by suppressing NF‐κB and MAPK activity	Ide et al. (2009)
	HCC	Retinoids and VitK2 cooperatively inhibit activation of the Ras/MAPK signaling pathway, the phosphorylation of RXRα protein, and the growth of HCC cells	Kanamori et al. (2007)
	HCC	VitK2 suppresses malignancy of HuH7 hepatoma cells via inhibition of connexin 43 promoter activity	Kaneda et al. (2008)
	HCC	VitK2 inhibits the growth of Smmc‐7721 cells by induction of apoptosis involving caspase 8 activation and p53. This apoptotic process was not mediated by the intrinsic apoptotic pathway	Li et al. (2010)
	HCC	VitK2 suppresses the proliferation of HCC cells by blocking the cell cycle G1/S progression through the transcriptional upregulation of p21 gene	Liu et al. (2007)
	HCC	VitK2 inhibits the growth of HCC cells via suppression of cyclin D1 expression through the IKK/IκB/NF‐κB pathway	Ozaki et al. (2007)
	HCC	VitK2 inhibits the growth and invasiveness of HCC cells via protein kinase A activation	Otsuka et al. (2004)
	HCC	VitK2 inhibits the NF‐κB activation through the inhibition of PKCα and ɛ kinase activities, and subsequent inhibition of PKD1 activation in human HCC cells	Xia et al. (2012)
	HCC	PKC‐δ enhances the HIF‐1α transcriptional activity by increasing the nuclear translocation, and VitK2 suppresses the HIF‐1α activation through the inhibition of PKC in HCC cells	Xia et al. (2019)
	HCC	The regulation of the HDGF gene expression is one of the crucial mechanisms of VitK2‐induced cell growth suppression for HCC	Yamamoto et al. (2009)
	Liver regeneration	VitK2‐enhanced liver regeneration is associated with oval cell expansion and upregulation of matrilin‐2 expression in 2‐AAF/PH rat model	Lin et al. (2014)
	Liver regeneration	Administration of VitK2 helped increase the proliferative ability of the hepatic oval cells in case of diabetes	Abdelhamid et al. (2019)
	Microglial activation & PD	VitK2 can directly suppress rotenone‐induced microglial activation by repressing ROS production and p38 activation	Yu et al. (2016)

	Neurite outgrowth	VitK1 and VitK2 enhance neurite outgrowth via the activation of PKA and MAPK‐mediated signaling pathways in PC12D cells	Tsang and Kamei (2002)
	Neuron toxicity	MK‐4 has the potential to protect neurons from methylmercury‐induced cell death, without increasing intracellular glutathione levels	Sakaue et al. (2011)
	Osteoblastogenesis & Osteoclastogenesis	MK‐4 stimulates osteoblastogenesis and inhibits osteoclastogenesis in human bone marrow cell culture	Koshihara et al. (2003)
	Osteogenesis	MK‐7 enhances vitamin D3‐induced bone development in human mesenchymal stem cells	Gigante et al. (2015)
	Osteoporosis	[1,25(OH)2D3] enhances VitK2 metabolic cycle functions in human osteoblasts	Miyake et al. (2001)
	Osteoporosis & Osteonecrosis	VitK2 could improve osteogenic differentiation via IL‐6/JAK/STAT signaling pathway	Wang et al. (2022)
	Osteoporosis & Osteonecrosis	VitK2 stimulates osteoblastogenesis and suppresses osteoclastogenesis by suppressing NF‐κB activation	Yamaguchi and Weitzmann (2011)
	Osteoporosis & Osteonecrosis	VitK2 has the potential to antagonize the effects of glucocorticoid on MC3T3‐E1 cells	Zhang et al. (2017)
	Oxidative cell injury & death	VitK1 and MK‐4 prevent oxidative cell death by blocking the activation of 12‐LOX and ROS generation	Li et al. (2009)
Photosynthetic system	MK acts as the secondary electron acceptor in type I homodimeric photosynthetic reaction center of *heliobacterium modesticaldum*	Kondo et al. (2015)
*In vitro & In vivo* (animal)	HCC	VitK2 and VitK3 were able to induce potent antitumor effects on HCC *in vitro* and *in vivo*, at least in part, by inducing G1 arrest of the cell cycle	Hitomi et al. (2005)
	Vascular calcification	VitK2 can suppress the expression of TLR2 and TLR4 and inhibit calcification of aortic intima in ApoE^−/−^ mice as well as smooth muscle cells	Wang et al. (2017)
		Sulfomenaquinone biosynthesis from MK requires only two genes, cyp128 and stf 3 in *Mycobacterium tuberculosis*	Sogi et al. (2016)
*In vivo* (animal)	Abdominal hernia	VitK2 can inhibit the expression of MMP‐2 and promote the increase of collagen expression to prevent abdominal hernia caused by collagen factors (rat)	Chen et al. (2015)
	AD & Mitochondrial dysfunction	Vitk2 is a mitochondrial electron carrier that rescues Pink1 deficiency and severe mitochondrial defects, resulting in more efficient ATP production	Vos et al. (2012)
	Aortic vascular smooth muscle cell calcification	MK‐4 reduces the mineralization and calcification of rat aortic vascular smooth muscle cells by regulating the BMP‐2 signaling pathway in order to attenuate the expression of Runx2.	Cui et al. (2018)
	Behavioral perturbations	25% reduction in the locomotor activity of dietary VitK‐deficient rats. In the radial‐arm maze assessment, a similar reduction in locomotor activity in the dietary VitK‐deficient rats with no alteration in performance (short‐term memory)	Cocchetto et al. (1985)
	Cognition	Lifetime consumption of a low‐VitK (VitK1 and MK‐4) diet resulted in cognitive deficits in the 20‐month‐old rats but did not affect cognition at 6 and 12 months of age, nor did it affect motor activity or anxiety at any age	Carrié et al. (2011)
	Liver regeneration	VitK2 can significantly improve the recovery of liver function after liver regeneration model in rats; when the dose is < 20mg/kg, the recovery of liver function is dose dependent	Zhang et al. (2013)
	Osteoporosis	VitK and D may have a synergistic effect on reducing bone loss (rat)	Matsunaga et al. (1999)
	Osteoporosis	MK‐4 appears to target osteoblasts, consequently inhibiting bone loss induced by ovariectomy (rat)	Asawa et al. (2004)
	Osteoporosis	VitK2 promotes bone healing in a rat femoral osteotomy model with or without glucocorticoid treatment	Iwamoto et al. (2010)

	Osteoporosis	The combined treatment with PTH1‐34 and MK‐4 may have a therapeutic advantage on bone healing around hydroxyapatite‐coated implants in osteoporotic rats	Li et al. (2018)
	Soft tissue calcification	A high dose of VitK2 can suppress experimental calcification of soft tissues induced by vitamin D2 (rat)	Seyama et al. (1996)
	Sphingolipids in brain	MK‐4 concentration was positively correlated with the concentrations of sulfatides and sphingomyelin, and negatively correlated with ganglioside concentration (rat)	Carrié et al. (2004)
	Valvular calcification & hypercholesterolemia	MK‐4 may have nonbeneficial effects on lipid levels, especially in the presence of hypercholesterolemia (mice)	Weisell et al. (2021)
	Vascular smooth muscle cell calcification	VitK2 inhibits rat vascular smooth muscle cell calcification by restoring the Gas6/Axl/Akt antiapoptotic pathway	Qiu et al. (2017)
	VitK level in myelin sulfatides	Dietary VitK1 was converted to MK‐4 in the brain. There were significant positive correlations between sulfatides and MK‐4 in the hippocampus and cortex. No significant correlations were observed in the striatum (rat)	Crivello et al. (2010)
	VitK level in tissues	The specific tissue distribution of VitK2 is affected not only by diet but also by gender, age, and the specific roles of VitK1, MK‐4, and MK‐6 in metabolism (rat)	Huber et al. (1999)
VitK reduction & hemostasis	NQO1 does not play a major role in the production of VitK hydroquinone suggesting the existence of multiple VitK reduction pathways (mice)	Ingram et al. (2013)
*Ex vivo*	Aortic calcification & apoptosis	VitK2 inhibits aortic calcification induced by warfarin via Gas6/Axl survival pathway (rat)	Jiang et al. (2016)
	Astrocyte dysfunction	MK‐7 protects astrocytes possibly by inhibiting mitochondrial dysfunction and the expression of proinflammatory cytokines. Gas6 may participate in these protective effects (rat).	Yang et al. (2020)
	Vascular smooth muscle cell calcification	VitK2 inhibits vascular smooth muscle cell calcification by restoring the Gas6/Axl/Akt antiapoptotic pathway. Gas6 is critical in VitK2‐mediated functions that attenuate CaCl2‐ and β‐GP‐induced VSMC calcification by blocking apoptosis (rat).	Qiu et al. (2017)
*In vitro In vivo* Human	Hepatic inflammation.	There was a beneficial effect of VitK supplementation on the management of hyperlipidemia‐associated inflammatory events via activating VitK‐dependent Gas6 protein.	Bordoloi et al. (2020)
Human	Aortic valve calcification	VitK supplementation slows down the progress of aortic valve calcification	Brandenburg et al. (2017)
	Arteriovenous fistula & Neointimal hyperplasia	VitK antagonists have detrimental effects on arteriovenous fistula remodeling, and VitK2 can reduce neointimal hyperplasia and calcification	Zaragatski et al. (2016)
	Bone health in postmenopausal women	MK‐4 helps improve hip bone geometry and bone strength in postmenopausal women by improving BMC and FNW, but it has little effect on DXA‐BMD	Knapen et al. (2007)
	Bone health in postmenopausal women	The suppression of serum levels of bone remodeling indices and the positive changes in lumbar spine BMD were observed in postmenopausal women following a 12‐month intervention period using a diet enriched with calcium, vitamin D, and VitK1 or MK‐7	Kanellakis et al. (2012)
	Bone health in postmenopausal women	Long‐term supplementation of MK‐7 may help postmenopausal women to prevent bone loss by significantly decreasing the age‐related decline in BMD and BMC at the lumbar spine and femoral neck, but not at the total hip	Knapen et al. (2013)
	Bone health in postmenopausal women	The use of melatonin, strontium (citrate), vitamin D3, and MK‐7 has a positive effect on the prevention or treatment of osteopenia, osteoporosis, or other bone‐related diseases	Maria et al. (2017)

	Bone health in postmenopausal women	For post‐menopausal or osteoporotic patients, there is no evidence that VitK affects BMD or vertebral fractures. It may reduce clinical fractures	Mott et al. (2019)
	Cardiovascular health for postmenopausal women	MK‐7 supplementation significantly decreased carotid‐femoral pulse wave velocity and stiffness Index β improves arterial stiffness in healthy postmenopausal women	Knapen et al. (2015)
	CKD	High plasma dp‐ucMGP level indicating a poor VitK status is a biomarker of kidney damage and cardiovascular risk in CKD patients. VitK2 supplementation may improve the carboxylation status of MGP	Kurnatowska et al. (2016)
	Cognition	A clinically and statistically significant association was seen between increased dietary VitK intake and better cognition and behavior among geriatric patients	Chouet et al. (2015)
	Coronary artery calcification	Gla protein is associated with coronary artery calcification and VitK status in healthy women. dp‐ucMGP may serve as a biomarker of VitK status	Dalmeijer et al. (2013)
	Coronary artery calcification	The benefits of MK‐4 supplementation were only observed in patients with VitK insufficiencies correlated with high PIVKA‐2 baseline levels, reducing brachial–ankle pulse wave velocity, but not coronary artery calcification	Ikari et al. (2016)
	Coronary calcification & atrial fibrillation	Patients using VitK antagonists show increased levels of coronary calcification	Weijs et al. (2011)
	Coronary calcification in postmenopausal women	High dietary menaquinones (MK‐4 to MK‐10), but probably not VitK1, intake is associated with reduced coronary calcification.	Beulens et al. (2009)
	COVID‐19	Early in acute COVID‐19, both VitK deficiency and vitamin D deficiency were independently associated with worse COVID‐19 disease severity	Desai et al. (2021)
	COVID‐19	Pneumonia‐induced extrahepatic VitK depletion could lead to accelerated elastic fiber damage and thrombosis in severe COVID‐19 due to impaired activation of MGP and endothelial protein S, respectively.	Dofferhoff et al. (2021)
	COVID‐19	Low VitK status was associated with mortality in patients with COVID‐19 in sex‐ and age‐adjusted analyses, but not in analyses additionally adjusted for comorbidities	Linneberg et al. (2021)
	HCC	Combined treatment of VitK2 and angiotensin‐converting enzyme inhibitor ameliorates hepatic dysplastic nodule in a liver cirrhosis patient	Yoshiji et al. (2007)
	Memory complaint	Increased dietary VitK intake was associated with fewer and less severe subjective memory complaint in older adults taking no VitK antagonists	Soutif‐Veillon et al. (2016)
	Osteoporosis in postmenopausal women	VitK2 plays a role in the maintenance and improvement of vertebral BMD and the prevention of fractures in postmenopausal women with osteoporosis	Huang et al. (2015)
Osteopenia in postmenopausal women	Treatment with MK‐7 as an add‐on to calcium and vitamin D increases the carboxylation of osteocalcin. But the treatment of postmenopausal women with osteopenia for 3 years did not affect biochemical markers of bone turnover, BMD, or bone microarchitecture	Rønn et al. (2021)

Abbreviations: 12‐LOX, 12‐lipoxygenase; AD, Alzheimer's disease; ATP, adenosine triphosphate; BMC, bone mineral content; BMD, bone mineral density; CKD, chronic kidney disease; Dp‐ucMGP, desphospho‐uncarboxylated‐MGP; FNW, femoral neck width; HCC, hepatocellular carcinoma; HDGF, hepatoma‐derived growth factor; HIF‐1α, hypoxia‐inducible factor‐1; MAPK, mitogen‐activated protein kinases; MGP, matrix Gla protein; MMP, matrix metalloproteinase; NQO1, NAD(P)H quinone oxidoreductase 1; PD, Parkinson's disease; PI3K, phosphatidylinositol 3‐kinase; PIVKA‐2, protein induced by VitK absence or antagonist‐2; PKC, protein kinase C; PTH, parathyroid hormone; ROS, reactive oxygen species; RXR, retinoid X receptor; TLR, Toll‐like receptor; VKDPs, vitamin K‐dependent proteins.

### 2.1 Promotion of osteogenesis and bone homeostasis

Bone formation and metabolism involve the nutrient calcium. Stomach acid converts ingested calcium to calcium ions, which are absorbed into the blood with the help of calcium transport proteins, driven by vitamin D3. Blood calcium ions subsequently enter the bone, regulated by calcitonin, and form the phosphate salt, hydroxyapatite, which gets deposited in the bone. The free calcium in hydroxyapatite is further electrostatically bound to osteocalcin (OC), one of the major noncollagenous VitK‐dependent proteins (VKDPs) found in the bones and secreted by osteoblasts, odontoblasts, and hypertrophic cartilage cells (Hauschka et al., 1989). Such binding must be promoted by VitK to establish the physiological osteogenesis process (bone mineralization). Deficiency in VitK would lead to the movement of free calcium from the bone to the blood again (resorption), resulting in osteoporosis (Maresz, 2015). However, deficiency of VitK is relatively unusual due to a continually recycled VitK usage process, or redox cycle, in our cells.

#### 2.1.1 Vitamin K redox cycle and activation of osteocalcin by Vitamin K

**FIGURE 3:**
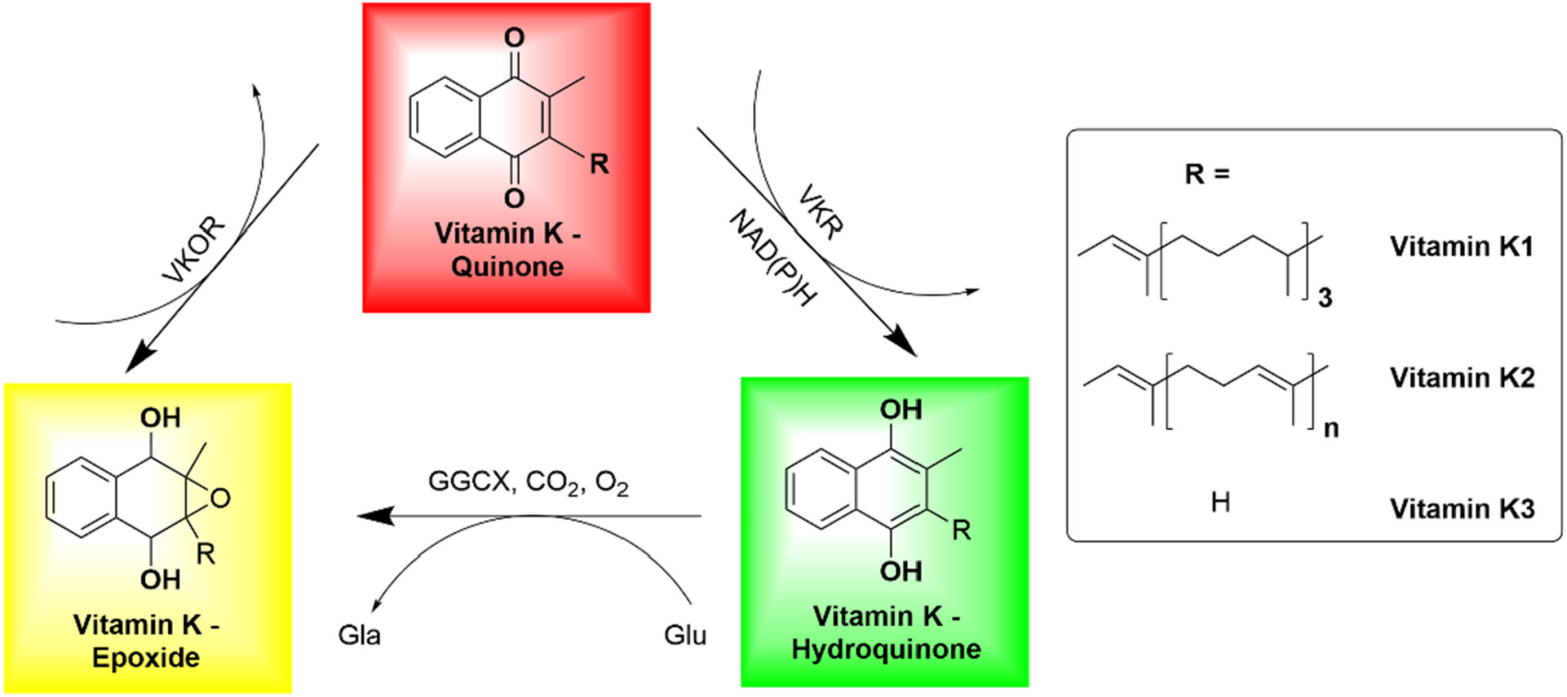
Vitamin K (VitK) redox cycle process. VitK‐dependent protein (VKDP) carboxylation starts with the reduction of VitK (quinone) to hydroquinone by VitK epoxide reductase (VKOR); during carboxylation, the glutamate (Glu) residue on VKDP is converted to γ‐carboxyglutamate (Gla) by γ‐glutamyl carboxylase (GGCX) with CO_2_ and O_2_ as cofactors, while hydroquinone is oxidized to vitamin K epoxide. VitK epoxide is transformed to hydroquinone by two‐step reduction by VKOR and yet‐to‐be‐defined VitK reductase (VKR), possibly NAD(P)H‐dependent reductase.

The major bone constituents are proteins (30%) in the organic part and hydroxyapatite (70%) in the inorganic part. The organic part contains at least two specific VitK‐dependent Gla proteins; OC and matrix Gla protein (MGP) (Azuma et al., 2014), which have calcium‐binding characteristics but need VitK as a cofactor to allow them to bind to calcium ions in hydroxyapatite (Shearer et al., 2012), which specifically transforms OC's/MGP's glutamate (Glu) residues to γ‐carboxyglutamate (Gla) residues through a VitK redox cycling process. This VitK cycle allows the body to reuse VitK, decreasing the dietary requirement (Inaba et al., 2015). This cyclic process is illustrated in Figure 3. Both VitK1 and VitK2 are involved in the activation of VKDPs, but long‐chain menaquinones show better bioactivity, suggesting that greater hydrophobicity correlates with higher bioavailability and a longer half‐life (Schurgers et al., 2010).

The carboxylation process of VKDPs begins with the conversion of the selective glutamate (Glu) residues on VKDPs to γ‐carboxyglutamate (Gla) residues by the γ‐glutamyl carboxylase (GGCX) enzyme, using as cofactors hydroquinone, CO_2_ and H_2_O. During this process, the reduced form of VitK undergoes a transition from the hydroquinone form to an oxidized state, the 2,3‐epoxide. The Gla residues dissociate, releasing two H^+^ and becoming negatively charged, enhancing their affinity for positively charged Ca^2+^ through electrostatic interactions, forming a hydroxyapatite lattice preceding bone mineralization and completing the physiological osteogenesis process. At the same time, after carboxylation, VitK epoxide is further reduced by two separate two‐electron reduction steps: to VitK (quinone) by VitK epoxide reductase, and to hydroquinone by a yet‐to‐be‐defined VitK reductase (Tie & Stafford, 2017), possibly a NAD(P)H‐dependent reductase (Ingram et al., 2013; Rishavy et al., 2013). Consequently, another Glu residue of VKDPs is carboxylated to Gla; ultimately into active OC being an osteogenic marker (Myneni & Mezey, 2017). Following this process, the majority of OC is accumulated in the bone matrix binding to calcium, while a small proportion of carboxylated‐OC (cOC) (~20%) flows into the blood circulation (Wei et al., 2018). So far, it is uncertain if there is a connection between cOC in blood and vascular calcification. It has also been reported that 1,25‐dihydroxyvitamin‐D [1,25(OH)_2_D_3_] can upregulate the activity of GGCX for OC production in humans and rats in a dose‐dependent manner (Karl et al., 1985; Miyake et al., 2001); conversely, a downregulation effect is seen in mice models (Wen et al., 2018). No clear explanation has been suggested so far for such observed differences in OC between human/rat and mice models.

Depending on the levels of carboxylation on the Glu sites, different forms of OC [cOC, uncarboxylated OC (ucOC) and undercarboxylated OC], with varying calcium‐binding affinities, can exist. However, the latter two forms are often not accurately referred to in most studies, probably due to limitations in their measurement (Li et al., 2018). ucOC or undercarboxylated OC is considered as a clinical indicator of VitK status, due to the dependency on VitK (Shiraki et al., 2010).

While contributing to bone formation by promoting calcium deposition, on the other hand, VitK2 can also prevent bone calcium dissolution and mobilization maintaining calcium homeostasis. Thus, positive effects of VitK2 on degenerative bone conditions, such as osteoporosis, have been suggested (Khalil et al., 2021).

#### 2.1.2 Regulation of osteoblasts and osteoclasts

Vitamin K2 also regulates the osteoblast function, including modulating the proliferation and differentiation of osteoblasts, inhibiting the induction of osteoblast apoptosis (Koshihara et al., 2003), and increasing the expression of osteogenic genes (Akbari & Rasouli‐Ghahroudi, 2018). Increased OC production (Matsunaga et al., 1999) and bone‐specific alkaline phosphatase activity (Asawa et al., 2004) were observed after exposure of osteoblasts to VitK2. Even in the case of glucocorticoid damage, VitK2 still exhibited an osteoprotective effect on osteoblasts (osteocyte density) and promoted bone healing in osteoporotic rat models (Iwamoto et al., 2010; Zhang et al., 2017). Some studies also showed that VitK2 (mainly as MK‐4 and MK‐7) can upregulate bone marker genes and extracellular matrix‐related genes, such as CYP3A4 and MSX2 (Akbari & Rasouli‐Ghahroudi, 2018), by activating the steroid and xenobiotic receptor (SXR), a nuclear receptor of osteoblasts modulating gene transcription and promoting collagen accumulation (Ichikawa et al., 2006).

On the other hand, activation of the transcription factor, nuclear factor kappa‐B (NF‐κB) signal pathway is essential for osteoclastogenesis. It was shown that VitK2 modulates osteoblast and osteoclast formation and activity via downregulation of basal and cytokine‐induced NF‐κB activation (Yamaguchi & Weitzmann, 2011). Many *in vitro* and *in vivo* studies have reported the suppressive effect of VitK2 on osteoclastogenesis and bone resorption, via various mechanisms: (i) stimulating expression of the cytokine osteoprotegerin (OPG) (an osteoclastogenesis inhibitory factor; Koshihara et al., 2003); (ii) reducing expression of the receptor activator of nuclear factor kappa‐B ligand (RANKL) on osteoblasts/osteoclasts, bound by the decoy receptor OPG (Yamaguchi & Weitzmann, 2011); (iii) limiting activities of bone‐resorbing factors, including prostaglandin‐E2 (Hara et al., 1995), interleukin (IL)‐1*α* (Akbari & Rasouli‐Ghahroudi, 2018), IL‐6 (Wang et al., 2022), and 1,25(OH)_2_D_3_ (Miyake et al., 2001; Poon et al., 2015).

**FIGURE 4:**
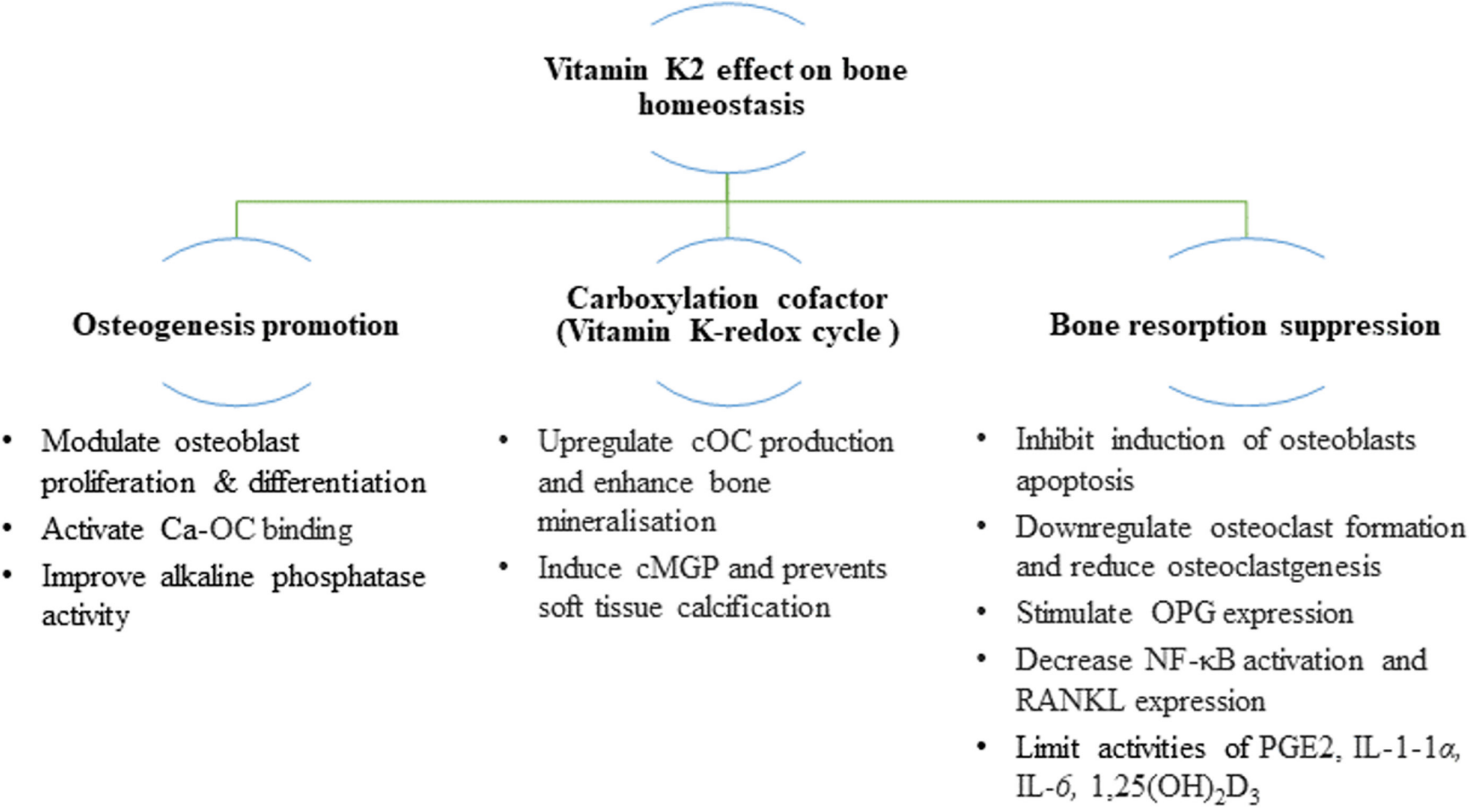
Biological effects of vitamin K2 on bone homeostasis through promotion of osteogenesis, suppression of bone resorption, and via activity as a carboxylation cofactor; OC: osteocalcin; MGP: matrix Gla protein; cOC: carboxylated OC; cMGP: carboxylated‐MGP; OPG: osteoprotegerin.

In summary, evidence supports the role of VitK2 in maintenance of bone health: (i) increasing bone strength and density, (ii) increasing bone mineral content, (iii) inhibiting bone resorption, (iv) decreasing fracture risk, (v) reducing urinary calcium loss, (vi) lowering serum alkaline phosphatase levels, and (vii) upregulating cOC and carboxylated‐MGP levels. This would suggest that VitK2 reduces bone calcium mobilization, increases bone calcium deposition, and strengthens bone construction. However, further investigations are required to support these observations. At the same time, VitK2 limits the occurrence of calcification in other organs due to reduced bone calcium loss (Maresz, 2015; Vermeer, 2012). Figure 4 presents a summary of the various effects of VitK on bone homeostasis.

### 2.2 Prevention of cardiovascular calcification

Ninety‐nine percent of bodily calcium is stored in bone, regulated in part by VitK2, with the remaining 1% circulating in the blood, muscle, and other tissues (Weaver, 2012). Low levels of VitK2 can cause disruption in binding between calcium and OC, leading to loss of calcium from bone and transportation of calcium to other tissues, which results in tissue calcification (Maresz, 2015). Therefore, calcification should be viewed as a standard part of the pathological aging process as the activity of various tissues or organs decreases, leading to functional decline (Roumeliotis et al., 2019). Microcalcifications have been seen in early intimal lesions of atherosclerotic human coronary arteries when considered healthy (Roijers et al., 2011). A notable organ is the pineal gland, which undergoes vigorous secretion in childhood and begins to decline in secretory function during puberty with significant calcification (Tan et al., 2018). Pineal calcification has long been reported in humans and is seen in nearly all adults leading to reduced melatonin secretion (Goree et al., 1963), which has also been suggested to be associated with Alzheimer's disease (AD) (Song, 2019). In addition, arterial blood vessels are lifelong active tissues, and arterial calcification has been observed in conditions with high atherogenic levels, such as diabetes, oxidative stress, and chronic kidney disease (CKD) (Roumeliotis et al., 2019). It is commonly seen in the aging population, with 96% of observed aortic and coronary artery calcification seen in people over the age of 70 (Lahtinen et al., 2015), causing arteriosclerosis and myocardial infarction, and accelerating mortality. It has also been recognized that cardiovascular calcification is an active and regulated process.

#### 2.2.1 Vascular calcification

Vascular calcification is a pathological process and is manifested by the extraosseous deposition of hydroxyapatite in the tunica media or tunica intima of the blood vessel walls (Wasilewski et al., 2019), with a consequent increased risk of hypertension, atheromatosis, ischemia, and myocardial infarction (Roumeliotis et al., 2019).

Emerging evidence from animal and clinical studies has shown that low VitK levels may be associated with vascular calcification and an elevated risk of cardiovascular diseases (CVDs). In women, an association with coronary artery calcification was seen (Dalmeijer et al., 2013). VitK2 can effectively stabilize mobile calcium, reduce artery calcium levels, inhibit calcium deposition in the blood vessel walls, and prevent the occurrence of CVDs (Beulens et al., 2009; Brandenburg et al., 2015; Kurnatowska et al., 2016). Relevant mechanisms of VitK2 involvement have been suggested to include promotion of bone calcification, prevention of bone calcium loss, and reduction in the source of deposited calcium (Gigante et al., 2015); MGP activation to inhibit the calcification of blood vessels in rat (Cui et al., 2018); growth‐arrest‐specific gene6 (Gas6) protein modulation to prevent vascular smooth muscle apoptosis and the calcification process; and other pathways to inhibit the calcification of the vascular wall in rat (Qiu et al., 2017).

##### 2.2.1.1 Calcification modulated by matrix Gla protein

Matrix Gla protein is the most influential natural inhibitor of all types of calcification in the body and is closely associated with metabolism, mortality, and CVD (Akbari & Rasouli‐Ghahroudi, 2018; Nishimoto & Nishimoto, 2005; Price et al., 1983). As a carboxylation substrate of VitK, MGP has been extensively studied as a central calcification inhibition protein in the development of calcification in the arteries, while facilitating normal bone metabolism. MGP carboxylation mediated by GGCX and VitK is as illustrated in Figure 3, and is followed by phosphorylation of the three serine residues of MGP (Roumeliotis et al., 2019). The phosphorylated MGP then attaches to hydroxyapatite crystals entering the blood vessel wall, forming a protein matrix on the surface of hydroxyapatite, which prevents their aggregation; thereby calcification is inhibited (Goiko et al., 2013). It has been reported that MGP expression in bone cells could be upregulated by vitamin D (Wen et al., 2018).

Conformational combinations of MGP are possible among various species in the body, depending on its carboxylation and/or phosphorylation state, such as phosphorylated‐carboxylated MGP (cMGP, the fully active form), dephosphorylated‐uncarboxylated MGP (dp‐ucMGP, the fully inactive form), dephosphorylated‐carboxylated MGP (dp‐cMGP), and phosphorylated‐uncarboxylated MGP (ucMGP) (Vermeer et al., 2016). Total uncarboxylated MGP (t‐ucMGP) includes dp‐ucMGP, but mainly with ucMGP (Dalmeijer et al., 2013). The exact role of MGP phosphorylation remains still unclear but it is believed to be the most crucial step during the MGP activation process. Roumeliotis et al. (2019) have examined current studies of the association between different MGP conformations and vascular calcification, renal function, mortality, and CVD. The ucMGP conformation maintains calcium‐binding capacity and maybe a vascular calcification prognostic biomarker but is not considered as a biomarker of VitK status (Chatrou et al., 2015; Epstein, 2016; Roumeliotis et al., 2019).

The fully inactive circulating conformation, dp‐ucMGP, is independently associated with (peripheral) vascular calcification and carotid femoral/aortic pulse wave velocity, suggesting that it might be a risk biomarker associated with mortality and CVD, allowing early intervention (Dalmeijer et al., 2013; Griffin et al., 2019; Wei et al., 2016). In a study with 40 patients undertaking orthopedic or abdominal surgery, higher plasma dp‐ucMGP levels were observed in patients with previous CVD history compared to those without, although no significant difference in dp‐cMGP was observed between groups. Postoperatively, the dp‐ucMGP concentration was significantly increased in the group with CVD history, and possible causes including nutritional defects were suggested (Dahlberg et al., 2018), thus highlighting the fact that VK intake may have in such situations. Currently, it remains to be elucidated whether OC and MGP would exhibit antagonistic or other interactions.

It has also been found that MGP is capable of downregulating bone morphogenetic protein‐2 (BMP‐2) and the transforming growth factor‐β function which results in vascular smooth muscle cell (VSMC) apoptosis (Li et al., 2008). There is evidence that such apoptosis promotes vascular calcification (El Asmar et al., 2014). It has been demonstrated that activated MGP, after binding with calcium and phosphate crystals, further activates arterial macrophages and upregulates phagocytosis of apoptotic bodies produced by VSMCs (under pathological conditions, such as in the case of long‐term vitamin K antagonist (VKA) drug treatment), thus avoiding ectopic calcification (Roumeliotis et al., 2019). VitK2 can also increase the transcription of MGP mRNA and tropoelastin mRNA, and inhibit osteopontin mRNA expression (Saito et al., 2007), thereby preventing osteo/chondrocytic transformation of blood vessel walls and restraining the vascular calcification process. It can also be seen that VitK2 carboxylates MGP in order to inhibit vascular calcification and in turn abolishes complications in CKD (Schurgers et al., 2010).

##### 2.2.1.2 Calcification modulated by Gas6

Vitamin K not only upregulates MGP expression as a calcification inhibitor in the cardiovascular system (Rennenberg et al., 2010) but also modulates the Gas6 pathway to inhibit the vascular calcification process. Gas6, another VKDP, is secreted from quiescent fibroblasts (Bellosta et al., 1997) and osteoblasts (Shiozawa et al., 2010). It is extensively expressed in the heart, lungs, intestine, kidney, brain, spleen, ovary, testis, bone marrow, VSMCs, macrophage, and liver (Perrier et al., 2010) and is the ligand for the Axl receptor tyrosine kinase (Axl) (Fernández‐Fernández et al., 2008). VitK‐dependent GGCX modulated‐carboxylation is also vital for the interaction of Gas6 with the Axl receptor. Gas6/Axl binding is known to regulate cell survival and prevent apoptosis and osteoblast‐like differentiation of endothelial cells and VSMCs (Lijnen et al., 2011). Therefore, Gas6 suppresses vascular calcification by inhibiting VSMC apoptosis; coronary heart disease and other CVDs are subsequently reduced (Beulens et al., 2009; Geleijnse et al., 2004; Vossen et al., 2015).

The association of VitK2 involvement in the repression of pathological vascular calcification and the upregulation of Gas6 expression has been shown (Jadhav et al., 2022; Jiang et al., 2016). In a study conducted by Qiu et al. (2017) Western blotting detected that substantial aortic smooth muscle Gas6 expression was restored after VitK2 treatment and that a significant decrease of VSMC calcification and apoptosis induced by CaCl_2_ and β‐sodium glycerophosphate was also observed. Additionally, it was noted that R428 (an Axl inhibitor) increased apoptosis and calcification even in the presence of VitK2 and Gas6, due to the significant blocking effect of R428 on the Gas6/Axl antiapoptotic pathway. In addition, Jiang et al. (2016) demonstrated decreased aortic calcification by VitK2 involving the Gas6/Axl survival pathway using a rat model of warfarin‐induced calcification. However, conversely, in a study using Gas6^−/−^ mice and Gas6^−/−^ derived VSMC, only minor calcification was observed, with no significant calcification upon depletion of Gas6 (Kaesler et al., 2016). The author suggested that Gas6 might not have a prominent role during the development of vascular calcification. There is also *in vivo* evidence showing that VitK2 inhibits intimal calcification of the aortic artery through suppression of Toll‐like receptor 2 (TLR2) and TLR4 expression (Wang et al., 2017).

In counterevidence, Weijs et al. (2011) showed that warfarin treatment caused increased levels of coronary artery calcification and that VitK2 could reverse such effects (Brandenburg et al., 2015). Subsequently, Zaragatski et al. (2016) also showed a powerful antagonistic effect of VitK2 in the treatment of arteriovenous intimal hyperplasia and calcification caused by warfarin‐induced CKD. Despite the belief that VitK2 may have value in inhibiting arterial calcification and the occurrence of CVDs, this has not been widely demonstrated. In 2016, in a 1‐year trial of 26 patients, coronary artery calcification progressed by 14% despite MK‐4 treatment, while a reduction in brachial–ankle pulse wave velocity was only seen in patients with baseline VitK insufficiency, with no change in arterial stiffness overall. However, trial limitations were reported, including small participant numbers and lack of a control group (Ikari et al., 2016).

#### 2.2.2 Cardiac valvular calcification

The atrioventricular and arterial valves are the structural foundations to ensure the unidirectional flow of cardiac blood. Several studies have shown that VKAs (i.e., warfarin) promote coronary artery calcification and valvular calcification (El Asmar et al., 2014; Weijs et al., 2011). It has also been reported that increased plasma dp‐ucMGP levels are associated with aortic valve calcification (Brandenburg et al., 2017). Thus, VitK2 supplementation has been suggested as a plausible intervention to inhibit the pathogenetic progression of these conditions (Brandenburg et al., 2015; Marquis‐Gravel et al., 2016). However, in a recent study using a hypercholesterolemic mouse model of calcific aortic valve disease, an MK‐4 diet did not beneficially impact aortic valve morphology but instead increased plasma levels of total cholesterol, triglycerides, and low‐density lipoprotein (Weisell et al., 2021).

#### 2.2.3 Cartilage calcification

Increasing evidence shows that osteoarthritis (OA), the most common form of degenerative joint disease, significantly correlates with progressive loss of articular cartilage due to calcification in articular cartilage, synovial fluid, or synovial membranes (Hawellek et al., 2016; Rafael et al., 2014). A relationship between OA and VitK deficiency has been suggested (Misra et al., 2013).

Gla‐rich protein (GRP) is the most recently identified VKDP and was first identified in sturgeon calcified cartilage (Shea et al., 2015). It is distributed primarily in bone, cartilage, skin, and vasculature, and possesses a high density of Gla residues (an estimated 15 in human), suggesting a strong calcium‐binding affinity (Viegas et al., 2015). GRP has recently been demonstrated to be directly associated with OA, through observing undercarboxylated GRP accumulation at sites of ectopic calcification in cartilage and synovial membranes of OA patients (Rafael et al., 2014). A similar association between OA and ucMGP/cMGP was also observed by Wallin et al. (2010) where an association between OA and carboxylation deficiency of both GRP and MGP was consistent. Thus, γ‐carboxylation modification is also believed to be vital for both GRP and MGP as calcification inhibitors, and a deficiency in carboxylation of these VKDPs may play an important role in causing OA. It has also been suggested that serum ucOC levels could be used as a biomarker of OA, based on a single‐arm clinical study of 25 Japanese patients with bilateral knee OA (Naito et al., 2012).

It has been suggested that GRP exhibits its calcification suppression effect via downregulation of osteoblast‐like differentiation of VSMCs, differentiation and maturation of osteoblasts, activity of alkaline phosphatase, and expression of osteogenic genes (Cavaco et al., 2016). It was reported that the expression of GRP in chondrocytes was inhibited by BMP‐2, which induces the osteogenic differentiation of VSMCs (Wen et al., 2018).

The occurrence of OA is also accompanied by tissue inflammation and associated with changes in collagen, levels of inflammatory mediators [i.e., matrix metalloproteinases (MMPs), IL‐8, monocyte chemotactic factor‐1], and related cartilage tissues induced by the deposition of calcium phosphate crystals (Nasi et al., 2016; Roemhildt et al., 2014). MMP‐2 hydrolyzes collagen and disintegrates the components of articular cartilage, resulting in cartilage damage. VitK2 inhibits the expression of MMP‐2, thereby promoting collagen expression and normalizing damaged soft tissue (Chen et al., 2015). In addition, VitK2 significantly suppressed experimental calcification of soft tissues of rats induced by vitamin D2 (Seyama et al., 1996). Moreover, GRP has been suggested to be involved in the cross talk between inflammation and cartilage calcification in OA, through observing the effects on calcification and inflammation in control and OA cells (Cavaco et al., 2016), which promotes GRP as a potential therapeutic target. However, it is worth noting that there are controversial results regarding the role of VitK2 in the maintenance of bone (and vascular) health, which are summarized in a recent review (Mandatori et al., 2021).

Recommendations regarding VitK intake differ in various countries, but a consistent conclusion arises: there may be certain synergistic and antagonistic effects between different VKDPs to maintain body homeostasis, and optimal VitK status is important to support homeostatic processes. Bone and the cardiovascular system are closely related during embryonic development, which may cause the differentiation of VSMCs into osteoblast‐like cells, secretion of BMP‐2, and other osteogenic proteins. Due to conflicting findings presented in the literature, further scientific research is needed to enhance the knowledge of the exact roles of VKDPs in bone and cardiovascular health.

### 2.3 Relief of menopausal symptoms

Hormonal and endocrine changes during menopause can greatly affect the reproductive system, bone density, and nervous and immune system function, among others. Osteoporosis, osteopenia, and increased calcification of abdominal aorta and carotid arteries in postmenopausal women have been noted in many studies (Nike et al., 2016; Wasilewski et al., 2019).

A meta‐analysis of 19 randomized, placebo‐controlled clinical trials encompassing 6759 participants showed that VitK2 plays an important preventive and therapeutic role for postmenopausal women with osteoporosis (Huang et al., 2015). Intake of MK‐4 daily for 3 years improved hip bone geometry and bone strength in postmenopausal women by increasing bone mineral content (BMC), but no increase in bone mineral density (BMD) was observed (Knapen et al., 2007); MK‐7 showed comparable effects in terms of improvement in both BMC and BMD (Knapen et al., 2013, 2015). Contrarily, no effects on BMD or vertebral fractures for postmenopausal or osteoporotic patients were concluded by a meta‐analysis (Mott et al., 2019); however, various factors, including trial methodology, measurement methods, VitK form and dosage used, and baseline population characteristics, were highlighted by the authors as potential reasons for the considerable heterogeneity among the trials included, and thus were limitations of the analysis. No improvement was seen in BMD, bone turnover, or bone microstructure after a 3‐year treatment with MK‐7 (375 μg/d) in a randomized, placebo‐controlled trial involving 142 postmenopausal women with osteopenia (Rønn et al., 2021), but a synergistic interplay of VitK2 with calcium (800 mg/d) and vitamin D3 (38 μg/d) was noticed, with the observation of increased osteocalcin carboxylation.

Vitamin K2 combined with vitamin D3, melatonin, and strontium also provided a significant regulating effect on osteoblastogenesis, BMD, bone turnover, and other indicators in the menopausal state, including a slowdown in the bone decline process, and provided both clinical and mechanistic support for the use of this combination for the prevention or treatment of osteopenia, osteoporosis, and other bone‐related diseases (Maria et al., 2017). A similar add‐on effect was observed in another clinical study (173 women between 55 and 65 years), where the combination treatment of VitK (100 μg/d), calcium (800 mg/d), and vitamin D3 (10 μg/d) significantly protected lumbar BMD compared to the control group, while the calcium/vitamin D3 combination did not (Kanellakis et al., 2012).

Furthermore, VitK2's health benefits have also been shown in other age‐related diseases (Simes et al., 2020). Specifically, Beulens et al. (2009) reported that high dietary intake of VitK2 was associated with significantly decreased coronary calcification from a study involving 564 postmenopausal women. The authors further proposed a less obvious decalcification effect from VitK1, which could possibly arise due to the difference in distribution of VitK1 and VitK2 in the body, where VitK1 is predominantly transported in the liver and less so in extrahepatic tissues resulting in less activation of MGP in the vasculature. In agreement with this result, a double‐blind, randomized, placebo‐controlled trial also showed that long‐term MK‐7 supplementation can significantly ameliorate arteriosclerosis and reduce aortic pulse wave velocity in healthy postmenopausal women, especially in women having high arterial stiffness (Knapen et al., 2015).

### 2.4 Hepatoprotection

The protective effects of VitK2 on liver regeneration after partial hepatectomy was studied by Zhang et al. (2013) who used the classical 2‐acetamido‐fluorene/partial hepatectomy model in Sprague–Dawley rats. In this study, VitK2 significantly increased serum albumin levels with concurrent reduction of the levels of alanine and aspartate aminotransferases, suggesting that VitK2 enhances liver regeneration. Further *in vitro* experiments suggested that such a liver regeneration effect could be related to the regulation of matrilin‐2 and hepatic oval cell proliferation (Lin et al., 2014; Abdelhamid et al., 2019). The combined treatment of VitK2 and an angiotensin‐converting enzyme inhibitor was found to clinically ameliorate a hepatic dysplastic nodule in a female patient (aged 66) with liver cirrhosis (Yoshiji et al., 2007). A recent study demonstrated the direct beneficial effect of VitK2 on the control of hyperlipidemia‐related hepatic inflammation through activating Gas6 carboxylation via arresting monocyte–hepatocyte adhesion (Bordoloi et al., 2020).

*In vitro* and *in vivo* experiments also demonstrated the cytotoxic effect of VitK2 on hepatocellular carcinoma (HCC) cells, with several underpinning mechanisms being reported (Xv et al., 2018; Yamamoto et al., 2009). It was suggested that VitK2 inhibits cell growth by downregulation of cyclin D1 expression through suppressing NF‐κB activation (IκB kinase (IKK)/IκB/NF‐κB pathway) (Ozaki et al., 2007) or through PKCα/NF‐κB and PKCɛ/PKD1/NF‐κB pathways (Xia et al., 2012), and suppression of HIF‐1α transactivation via inhibiting PKC‐δ (Xia et al., 2019). It has also been proposed that the inhibitory activity of VitK2 on HCC cell proliferation is associated with upregulation of the cell‐cycle regulatory protein p21 transcription (Liu et al., 2007), downregulating hepatoma‐derived growth factor expression (Yamamoto et al., 2009), as well as activating PKA (Otsuka et al., 2004), causing subsequent G1 cell‐cycle arrest (Hitomi et al., 2005). Thus, VitK2 inhibited HCC cell growth by suppressing the expression of cyclin D1 via the IKK/NF‐κB pathway and, therefore, could be a useful strategy in treating HCC.

**FIGURE 5:**
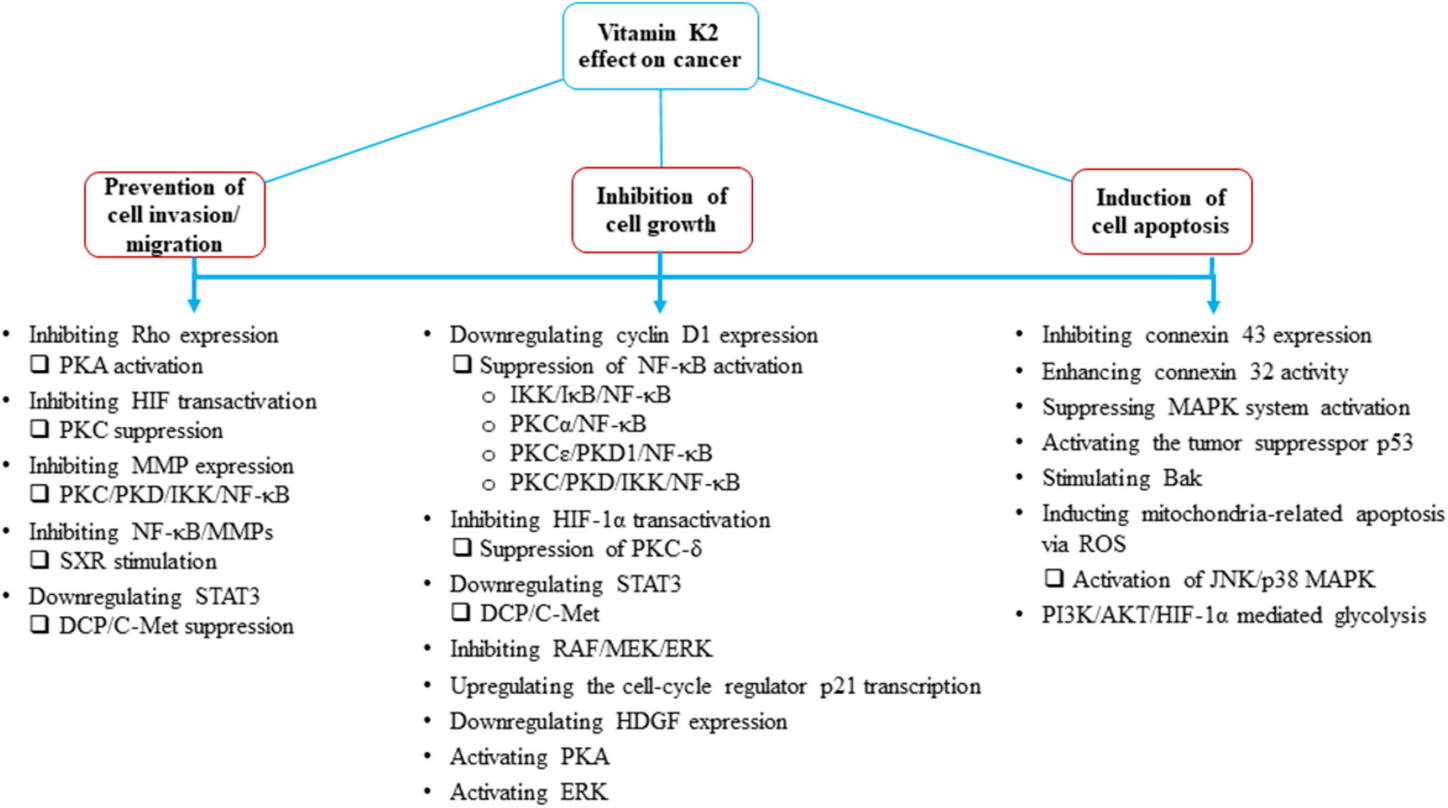
A mechanistic overview of proposed vitamin K2 toxicity in various cancer cells, including lung, pancreatic, bladder, and leukemia. RhO: Ras homologous; PKA: protein kinase A; HIF: hypoxia‐inducible factor; PKC: protein kinase C; PKD: protein kinase D; IKK: IκB kinase; NF‐κB: nuclear factor kappa‐B; MMP: matrix metalloproteinase; SXR: steroid and xenobiotic receptor; STAT: signal transducer and activator of transcription; DCP: Des‐gamma‐carboxy prothrombin; MEK: mitogen‐activated extracellular signal‐regulated kinase; ERK: extracellular signal‐regulated kinase; HDGF: hepatoma‐derived growth factor; Bak: Bcl‐2 antagonist killer 1; MAPK: mitogen‐activated protein kinase; ROS: reactive oxygen species; JNK: c‐Jun N‐terminal kinase; PI3K: phosphoinositide 3‐kinase; AKT: serine–threonine kinase.

In addition, VitK2 also induces differentiation and apoptosis of HCC cells. For instance, VitK2 suppressed the malignancy of cultured HuH7 cancer cells and promoted a normal hepatocyte phenotype via inhibiting connexin 43 expression and enhancing connexin 32 activity, respectively (Kaneda et al., 2008). The apoptotic effect of VitK2 has also been suggested to be associated with the mitochondrial pathway (Xv et al., 2018) involving inhibition of MAPK system activation (Kanamori et al., 2007), and the extrinsic apoptosis pathway involving activation of p53, a tumor‐repressor gene (Li et al., 2010). Furthermore, a preventive effect of VitK2 on tumor invasion was also reported in HCC cell lines via inhibition of MMP expression (Ide et al., 2009). The effects of VitK2 in various (i.e., lung, pancreatic, bladder, and leukemia) cancer cells through multiple signaling pathways are presented in Figure 5.

In summary, supplementation of the recommended dose of VitK2 may provide a positive effect on the prevention and treatment of malignant tumors. Since the mechanisms we have described so far are only part of the complete signaling network regulated by VKDPs, the controversy over the role of some proteins in various cancers would require further investigation. Some studies have yielded promising results, but there was a lack of focus on the specific carboxylated forms of VKDP when carrying out specific measurement and comparison of malignant tumors; therefore, a more comprehensive and unified comparative analysis cannot be performed in this review. In our view, an appropriate strategy or guideline for improving VitK2 status in patients with liver diseases should be established, as current recommendations might not be optimal.

### 2.5 Enhancement of mitochondrial energy release

**FIGURE 6:**
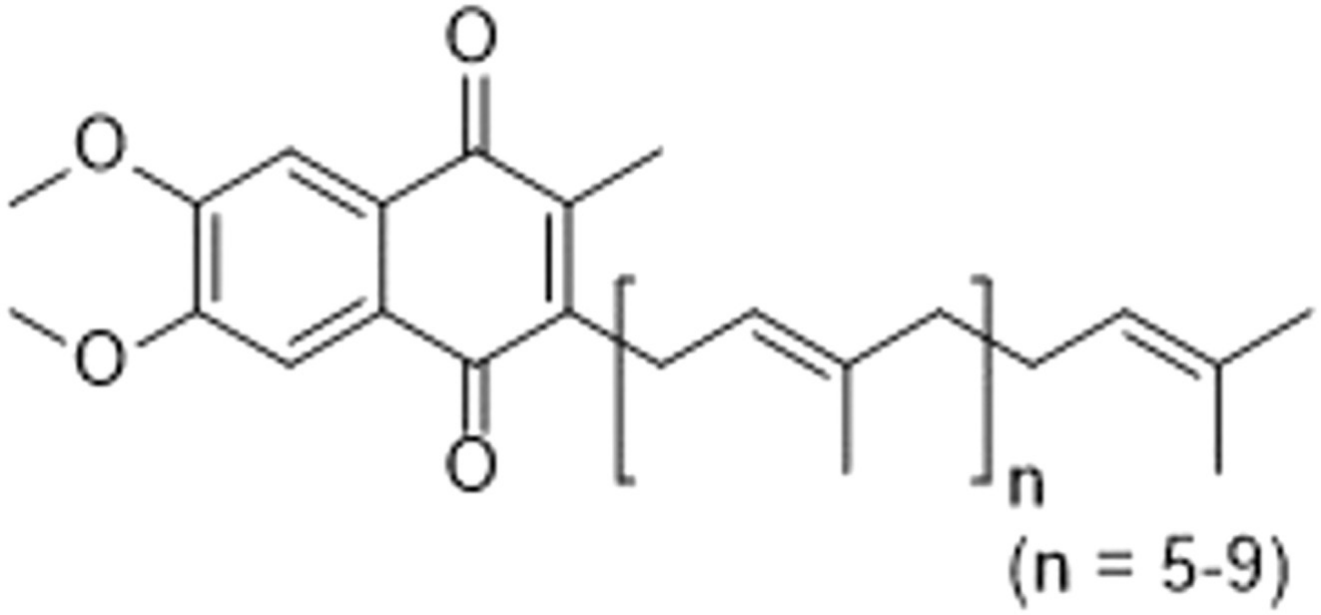
Chemical structure of CoQ_10_ (ubiquinone, *n* = 9).

Mitochondria are found in the cytoplasm of almost all eukaryotic cells. They use ubiquinones acting as electron carriers in the respiratory chain to produce adenosine triphosphate (ATP) and regulate cellular energy metabolism. Different forms of ubiquinones are found in various organisms, with their cellular location being mainly in the inner mitochondrial membrane. Coenzyme Q_10_ (CoQ_10_) is an essential VitK‐like substance with a naphthoquinone ring and 10 isoprene units (Figure 6). It is the most common ubiquinone in humans (Kurosu & Begari, 2010). Functions of CoQ_10_ as a component of the mitochondrial respiratory chain are well established. Based on the electron transport effects of menaquinones and their structural similarity to CoQ_10_, albeit being less lipophilic, it has been hypothesized that VitK2 treatment of mitochondrial diseases could be advantageous, especially under hypoxic conditions, when the cytochrome‐c‐oxidase system cannot function normally. It could potentially be of some significance to ischemic cells in cases of stroke and myocardial infarction.

Menaquinones also play an essential part in energy generation in prokaryotes, associated with their role in active electron transport, especially for Gram‐positive bacteria under either aerobic or anaerobic respiration, such as *Mycobacterium* spp. In Gram‐negative organisms, ubiquinones are used in aerobic respiration processes, whereas menaquinones are utilized under anaerobic conditions (Suvarna et al., 1998). Even in heliobacteria, VitK2, specifically the quinone unit, plays the role of the secondary electron acceptor in the process of successful photosynthesis (Kondo et al., 2015).

When glucose is broken down in the mitochondria (eukaryotes)/in the cell membrane (prokaryotes), electrons are released from hydrogen and transported along the membrane by ubiquinones/menaquinones to oxygen, with the help of the enzymes in the respiratory chain, such as the cytochrome‐c‐oxidase system. Thus, water molecules are formed, causing protons to be pumped across the membrane, with ATP energy released at the same time (Pamplona, 2011). The tautomerization between the core skeleton naphthoquinone and the naphthol structure in ubiquinones/menaquinones is considered to play a critical role in the electron transport system for various observed protective effects (Ivanova et al., 2018).

One published study attributed VitK2 as serving as a mitochondrial electron carrier and rescuing mitochondrial dysfunction due to Pink1 protein deficiency in the multicellular eukaryote *D. melanogaster* (Vos et al., 2012). This suggests that VitK2 has the potential to treat mitochondrial‐associated diseases or defects in ubiquinone biosynthesis. Pink1 is a protein kinase located in the outer membrane of the mitochondria. It has the function of protecting mitochondria when the cell has abnormally high energy requirements. When mitochondria are damaged, the cell recognizes and clears the dysfunctional mitochondria through the Pink1 protein (Vos et al., 2012) and/or mitochondrial quality‐control loop (Tang et al., 2022). It is now known that this steady‐state imbalance is related to the occurrence of Parkinson's disease (PD) (Opdebeeck et al., 2019). Such a result indicates a molecular mechanism of VitK2 in the treatment of PD. However, in a 2019 study using human CoQ_10_‐deficient cell lines and yeast carrying mutations in genes required for CoQ_6_ biosynthesis, Cerqua and coauthors claimed that VitK2, despite reaching mitochondria, restored neither electron flow in the respiratory chain nor ATP synthesis (Cerqua et al., 2019). It was considered that the role of VitK2 as electron carrier (if confirmed) might probably be restricted to *Drosophila*, rather than being a general phenomenon in eukaryotic cells.

In recent years, because of the lack of VitK2 biosynthesis enzymes in humans and the vital role played by VitK2 in bacteria, the design of inhibitors targeting the VitK2 biosynthetic pathway has received considerable attention. This is especially so for the design of novel and selective antimicrobial agents targeting multidrug‐resistant Gram‐positive pathogens including *Mycobacterium tuberculosis* (Sogi et al., 2016).

### 2.6 Neuroprotection

Oxidative and neuroinflammatory mechanisms of cellular damage are associated with many neurological disorders, including neurodegenerative conditions such as Alzheimer's disease (AD) and Parkinson's disease (PD). An increasing body of evidence suggests the possible role of VitK supplementation as a novel neuroprotective strategy in the maintenance of nerve integrity and normal brain function, including cognition and behavior (Carrié et al., 2011; Chouet et al., 2015; Cocchetto et al., 1985; Ferland, 2013; Josey et al., 2013).

Modulatory roles of VitK in cognition and behavior and in sphingolipid homeostasis have been supported by a growing body of preclinical evidence. Following a 6‐month treatment with a VitK1 diet, rats had increased MK‐4 levels in various brain regions, with significantly higher MK‐4 levels in myelinated regions (the pons medulla and midbrain; Carrié et al., 2004). The same research group also revealed that long‐term VitK1 and MK‐2 depletion correlated with increased cognitive impairment, as measured by the Morris Water Maze (MWM) test in rat models, especially in older animals (20 months), who took longer to perform the task (Carrié et al., 2011). In addition, this observation was further supported by human studies (Chouet et al., 2015; Soutif‐Veillon et al., 2016).

In a behavioral‐perturbations study, Cocchetto et al. (1985) observed a 25% reduction in locomotor activity in a rat group fed a VitK‐deficient diet compared with that of the control group in the open‐field paradigm assessment. Significantly less exploratory behaviors were also seen in the group receiving warfarin treatment. The radial‐arm maze assessment provided similar results in terms of reduction in locomotor activity by VitK dietary depletion, but no alteration in short‐term memory was evident.

Several studies have provided evidence that MK‐4 is the predominant form of VitK in both rat and human brain tissue (Ferland et al., 2016; Nakagawa et al., 2010), even though the majority of extrahepatic tissues have VitK1 and MK‐4 (Ferland, 2012). MK‐4 was reported to account for >98% of the total VK content in the rat brain between 6 and 21 months (Carrié et al., 2004). Sex, age, and diet also influence the concentration of MK‐4 in brain (Ferland et al., 2016). Higher MK‐4 levels in the cortex and cerebellum of female Brown Norway rats were observed compared to males on a similar diet. Interestingly, the concentration of MK‐4 reduced from 12 to 24 months (Huber et al., 1999).

It is well known that VitK is a necessary factor for the biosynthesis and metabolism of sphingolipids, by modulating certain key enzyme activities involved (Lev, 1979). Sphingolipids represent an important class of lipids, which exist in high concentrations in neuronal and glial cell membranes and function in brain cell events, including signaling, proliferation, differentiation, survival, synaptic transmission, neuronal–glial interaction, and myelin stability (Olsen & Færgeman, 2017). The major sphingolipids, including ceramide, sphingomyelin, cerebroside, sulfatides, and gangliosides (Ferland, 2012), are associated with neuroinflammation and neurodegeneration because of their effects on microglial activation and accumulation of amyloid precursor protein (Alisi et al., 2019). In rat experiments conducted by Carrié et al. (2004), MK‐4 levels were found to be positively correlated with concentrations of sphingomyelin and sulfatides, and negatively correlated with ganglioside concentration in both low (80 μg/kg) and adequate (500 μg/kg) VitK1 diet groups. Crivello et al. (2010) also showed significant positive correlations between sulfatide and MK‐4 levels in the hippocampus and cortex of 12‐ and 24‐month male Fisher rats (*n* = 344) receiving VitK1 dietary supplementation, but no significant correlations were found in the striatum.

Due to the wide distribution of Gas6 in the central and peripheral nervous systems, the neurofunctions of Gas6 to maintain adequate cerebral homeostasis have been studied, including antiapoptotic, cell growth, mitogenic, myelinating activity in neuronal and glial cells (especially oligodendrocytes, Schwann cells, and microglia) (Ferland, 2012). Since Gas6 is a VKDP, it has been suggested that VitK2 must play an important protective role in the nervous system by regulating Gas6. Huang et al. (2021) suggested that VitK2's protective effect against amyloid β‐protein (Aβ) cytotoxicity in neural cells might be through Gas6 activation, leading to upregulation of the phosphatidylinositol 3‐kinase (PI3K)/Akt/Axl pathway and inhibition of caspase‐3‐mediated apoptosis. Aβ plaque accumulation in the brain is clinically considered as a biocharacteristic feature of AD, being responsible for the massive neuronal death seen. Specifically, Hadipour et al. (2020) recently demonstrated that VitK2 protected PC12 cells against Aβ_(1‐42)_ and H_2_O_2_‐induced apoptosis in a model of AD cell damage, via inactivating the p38 MAPK pathway. Similarly, Tsang and Kamei (2002) showed that both MK‐4 and VitK1 enhanced neurite outgrowth in PC12D cells by activation of protein kinase (PK) A and MAPK‐mediated signaling pathways. The neurofibrillary tangles of the microtubule‐binding protein tau are another suggested mechanism defining AD (Alisi et al., 2019). Protein S has a neuronal protective role during cerebral ischemia and hypoxic injury (Ferland, 2012) both of which contribute to vascular dementia caused by several vascular pathologies (Alisi et al., 2019).

**FIGURE 7:**
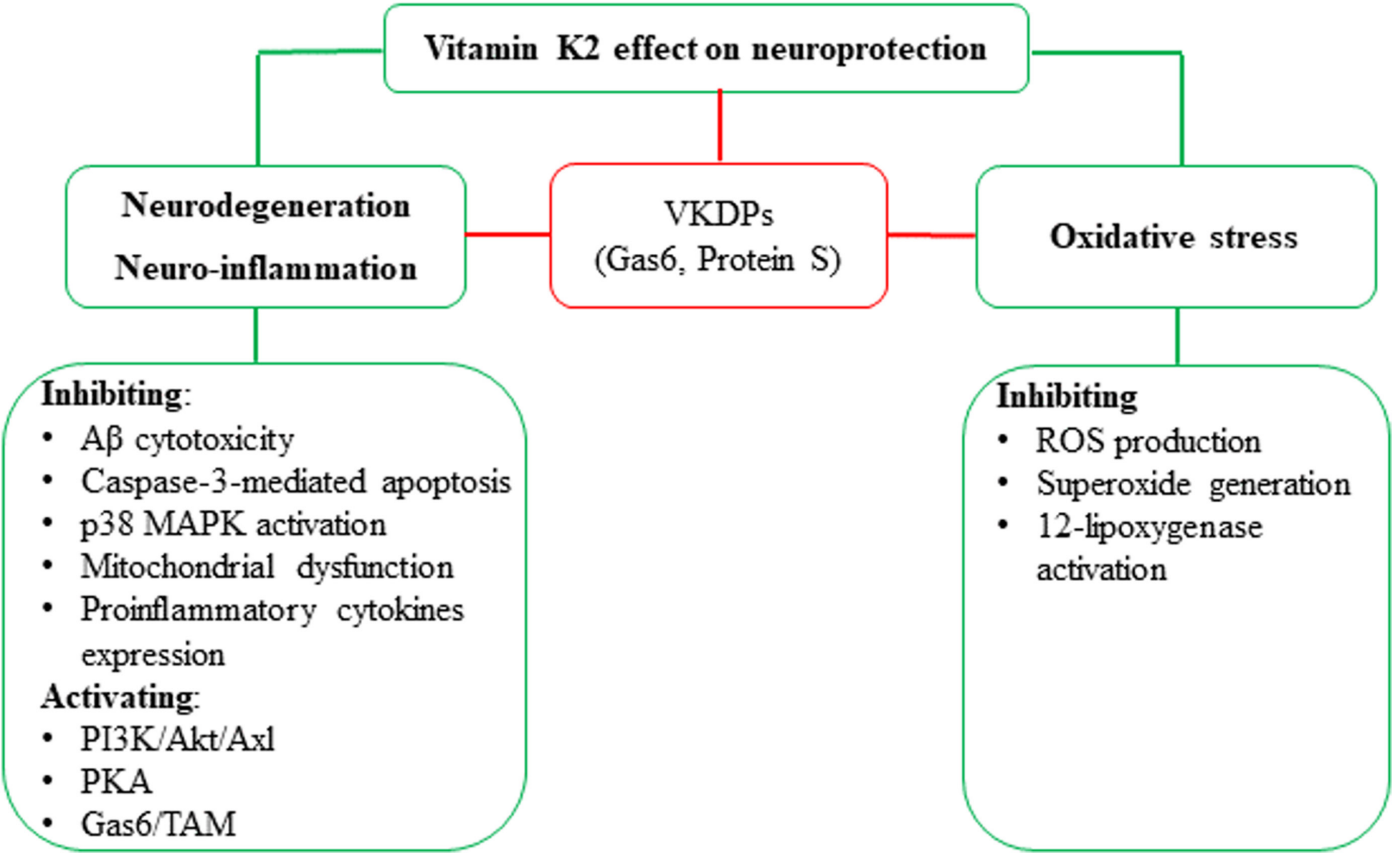
The protective effect of vitamin K2 on neuroprotection. It is considered to be associated with modulating neurodegeneration, inflammation, and oxidative stress via with (green route) or without (red) the involvement of VKDPs (Gas6, Protein S). VKDP: vitamin K‐dependent protein; Gas6: growth‐arrest‐specific gene 6; Aβ: amyloid β‐protein; ROS: reactive oxygen species; MAPK: mitogen‐activated protein kinase; PI3K: phosphatidylinositol 3‐kinase; Akt: protein kinase B; Axl: Axl receptor tyrosine kinase; PKA: protein kinase A; TAM: Tyro3, Axl, and MERTK.

In the same vein, Yang et al. (2017, 2020) reported that VitK2 treatment, particularly MK‐7, exhibited a significant neuroprotective effect on cultured rat astrocytes against hypoxia‐induced apoptosis, and proposed the possible involvement of protein S and Gas6 by inhibiting mitochondrial dysfunction and the expression of proinflammatory cytokines, and/or reducing reactive oxygen species (ROS) as well as superoxide generation. A placebo‐controlled clinical trial (DRKS00019932) is currently ongoing to determine the potential effects of MK‐7 in patients with PD and mitochondrial dysfunction, where 130 participants are involved (Prasuhn et al., 2021). The proposed actions of VitK's neuroprotective effects are summarized in Figure 7.

A limited number of studies have provided evidence of a direct action of VitK (primarily VitK2), where VKDP may not be involved. In both primary cultured neurons and human neuroblastoma IMR‐32 cells, MK‐4 and VitK1 inhibited methylmercury‐induced neuronal death without affecting glutathione levels (Sakaue et al., 2011), suggesting the potential of VitK treatment for neural disease conditions involving glutathione depletion. Evidence also indicates that VitK2 directly suppresses rotenone‐induced activation of cultured microglial BV2 cells by inhibiting ROS production of reactive oxygen species and p38 MAPK activation (Yu et al., 2016), and by inhibiting the activation of 12‐lipoxygenase in developing oligodendrocytes to prevent oxidative cell death (Li et al., 2009).

### 2.7 Coronavirus disease

**FIGURE 8:**
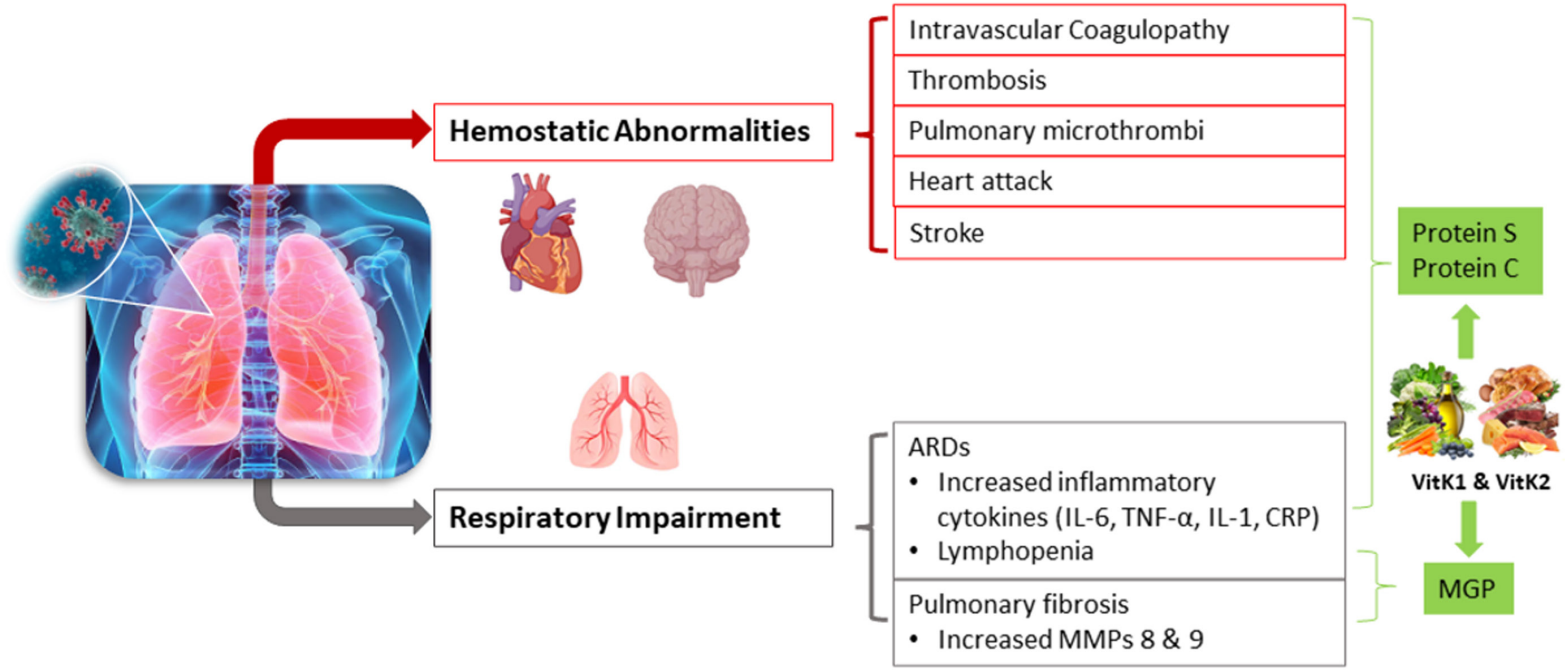
The potential modulating role of vitamin K (VitK) in thromboembolism and respiratory impairment related to COVID‐19 pathogenesis. Following the entry of SARS‐CoV‐2 into alveolar type II cells, the virus causes lung epithelial cell and endothelial cell infection. The former leads to respiratory impairment, associated with (i) the production of pro‐inflammatory cytokines [i.e., Interleukin 6 (IL‐6), Tumor Necrosis Factor‐alpha (TNF‐α), IL‐1, and C‐reactive protein (CRP)], causing acute respiratory distress syndrome (ARDS), and (ii) increased levels of metalloproteinases (MMPs) 8 and 9, inducing pulmonary fibrosis (grey route). The latter affects normal coagulation processes, resulting in hemostatic abnormalities including intravascular coagulopathy, thrombosis, pulmonary microthrombi, heart attacks, and stroke (red route). This pathogenesis is associated with insufficient carboxylation of Matrix Gla protein (MGP), Protein S, and Protein C; therefore, a modulating role of VitK is proposed (green route).

A recent review has collected evidence exploring the potential beneficial role of VitK in the pathogenesis of COVID‐19 through proposed effects on coagulation and/or immuno‐regulation (Figure 8; Kudelko et al., 2021). By using a causal loop diagram, Goddek proposed a potential synergistic effect between VitK and vitamin D against COVID‐19, to prevent long‐term health risks (Goddek, 2020). This was demonstrated by a prospective observational study involving 100 COVID‐19 patients and 50 controls (median age 55), where worse disease severity was found to be positively and independently correlated with both VitK and vitamin D deficiency using the dp‐ucMGP level as a biomarker (Desai et al., 2021), but the clinical study is lacking in order to prove whether optimization of VitK and vitamin D status could have such positive impact clinically.

Chiodini et al. (2021) very recently published a meta‐analysis based on 54 studies, showing that COVID‐19 patients requiring hospital admission and with low vitamin D levels present an increased risk of respiratory distress and mortality due to respiratory failure or other complications. This supports the protective role of administration of vitamin D against acute respiratory tract infection based on its anti‐inflammatory properties as previously suggested (Martineau et al., 2019). However, pro‐calcification effects of vitamin D in COVID‐19 patients have been hypothesized, based on a recent study with 135 hospitalized patients, where vitamin D sufficient patients (25(OH)D >50 nmol/L) had increased degradation of elastic fiber in lung compared to those with mild deficiency (25(OH)D 25–50 nmol/L); whereas no difference in vitamin D level was seen between patients in good and bad (intubation and/or death) condition (Walk et al., 2020). This suggests that VitK might be considered when administration of calcium is needed for COVID‐19 patients, in order to compensate for the potential negative consequence of fiber degradation caused by calcification. Similar findings have been further demonstrated in human trials with measurement of VitK alone, including a trial with 138 COVID‐19 patients and 138 controls, where it was found that an elevated dp‐ucMGP level was associated with mortality in patients, after adjusting for gender and age factors (Linneberg et al., 2021). These data so far suggest that there might be a pathway of pneumonia‐induced extrahepatic VitK depletion in COVID‐19 patients resulting in accelerated elastic fiber degradation and thrombosis formation, due to impaired activation of VKDPs (MGP and Protein S; Dofferhoff et al., 2021). A randomized, controlled phase‐2 trial has recently been set up to investigate effects of VitK2 (MK‐7) in COVID‐19 (NCT04770740) (Hospital, 2021). More immediate observational studies and larger randomized clinical trials are warranted to further evaluate the effect of VitK on the prognosis of COVID‐19 patients and the beneficial impact of VitK supplementation on clinical severity.

In the meanwhile, we further suggest that monitoring and supplementation of VitK2 (or even VitK in general), based on recommended adequate daily doses of 90 mg (female >19 years old)/120 mg (male >19 years old) (NIH, 2021), could also be considered as a part of the COVID‐19 treatment strategy in counteracting COVID‐19 infection and reducing complications; thus, reinforcing the health maintenance and therapeutic potential of optimal circulatory levels of VitK. It is also recommended that more investigations using other biomarkers (rather than dp‐ucMGP) to measure the VitK2 level are needed to provide more robust scientific evidence.

## 3 CONCLUSION

Vitamin K2, together with VitK1, is one of the important and essential naturally occurring vitamins for human health. This study provides a comprehensive overview of available data to add to the understanding of its biological ‘fingerprint’, and maps the effects of VitK2, extending from its well‐known role in blood coagulation to promotion of osteogenesis, prevention of calcification, relief of menopausal symptoms, enhancement of mitochondrial energy release, and its protective effects on the liver and nerves. It is clear that VKDPs are a primary target for VitK2, such as OC, MGP, Gas6, and Protein S, thus controlling the body's various physiological functions by regulation of Ca^2+^ distribution. Mitochondrial respiratory chains have been considered to be the second target of VitK2 in energy conversion and release, as well as cell survival through the electron transfer process. But there is currently limited proof for this claim, especially for mammalian cells. Taken together, according to current studies, VitK, especially VitK2, is an important bioactive nutrient family for human health. The beneficial effects of VitK2 in various conditions emphasize the importance of VitK2 in the global diet, as evidenced by the results presented in this review. However, biological and clinical data are still inconsistent and conflicting; therefore, more in‐depth investigations, including larger and longer duration trials with better design are warranted to optimize its use as a potentially useful strategy for the prevention and treatment of a range of disease conditions.

## FUNDING INFORMATION

This research received no external funding.

## CONFLICT OF INTEREST

The authors declare no conflict of interest.

## Data Availability

There is no primary data associated with this manuscript.
