# Correction to “The Biological Responses of Vitamin K2: A Comprehensive Review”

**DOI:** 10.1002/fsn3.70005

**Published:** 2025-01-27

Yan, Q., Zhang, T., O'Connor, C., Barlow, J. W., Walsh, J., Scalabrino, G., Xu, F., & Sheridan, H. (2023). The biological responses of vitamin K2: A comprehensive review. *Food Science & Nutrition*, 11, 1634–1656. https://doi.org/10.1002/fsn3.3213.

In the last paragraph of section 2.7 (Coronavirus disease), there are errors in the units in the following text: “…based on recommended adequate daily doses of 90 mg (female > 19 years old)/120 mg (male > 19 years old) (NIH, 2021).”

This should have read: “…based on the recommended adequate daily doses of 90 μg (female > 19 years old)/120 μg (male > 19 years old) (NIH, 2021).”

We apologize for this error.

